# The treatment of hypertension in people with dementia: a systematic review of observational studies

**DOI:** 10.1186/1471-2318-14-19

**Published:** 2014-02-12

**Authors:** Tomas J Welsh, John R Gladman, Adam L Gordon

**Affiliations:** 1 Division of Rehabilitation and Ageing, School of Medicine, B99, B Floor, University of Nottingham, Nottingham NG7 2UH, UK

**Keywords:** Hypertension, Dementia, Systematic review, Treatment, Antihypertensive

## Abstract

**Background:**

Hypertension is very common in older people and a number of trials of antihypertensives have demonstrated benefit from treatment in even the oldest old. However, people with dementia were significantly under-represented in these studies and as a population are more likely to be physically frail, to suffer orthostatic hypotension and to experience adverse effects from polypharmacy at a lower drug count. It may be that different thresholds for commencement and cessation of treatment should be considered and may already be used for this group. Against this background this review sets out to describe the prevalence of hypertension in people with dementia, its treatment, change in treatment over time and the achievement of blood pressure (BP) control.

**Methods:**

The PubMed, Cochrane, Embase and PsychINFO databases were searched for observational studies involving people with dementia and a diagnosis of hypertension. The search was limited to English language articles involving adults and humans published from 1990 onwards. Abstracts and titles were then reviewed with eligible articles read in full. Bibliographies were examined for further relevant studies. The final selection of studies was then analysed and appraised.

**Results:**

Thirteen articles were identified for analysis. The prevalence of hypertension in people with dementia was 45% (range 35%-84%). 73% of these were on at least one antihypertensive, with diuretics being the most common. The reported prevalence of hypertension in study populations remained unchanged over time. ACEi/ARBs and calcium channel blockers were prescribed more frequently in more recent studies whilst use of β-blockers and diuretics remained unchanged. Target blood pressure was achieved in 55% of those on treatment.

**Conclusion:**

Hypertension is as common in people with dementia as in other populations and is as commonly treated with antihypertensive drugs. The findings presented here will support further work to establish the risk-benefit of antihypertensive treatment in patients with dementia and, if differing ratios are identified, to establish dementia-specific guidelines for management.

## Background

Hypertension is common in very old people, approximately 80% of those aged over 80 are hypertensive [1], and remains a risk factor for cardiovascular and cerebrovascular disease in later life. A number of trials of antihypertensive medication, most notably the Hypertension in the Very Elderly Trial (HYVET) [2], the Systolic Hypertension in Europe Study (Syst-Eur) [3], the Systolic Hypertension in the Elderly Program (SHEP) [4] and the Study on Cognition and Prognosis in the Elderly (SCOPE) [5], demonstrated that antihypertensives can benefit the oldest old. A Cochrane review of the treatment of hypertension in older adults confirmed that treatment reduced cardiovascular events, but also showed that it was associated with a significant increase in the rate of treatment withdrawal [6].

The findings of these trials have been incorporated into many widely accepted guidelines for the treatment of hypertension, such as those of the Joint National Committee on Prevention, Detection, Evaluation, and Treatment of High Blood Pressure (JNC), which have promoted increasingly aggressive targets for blood pressure control in older people [7,8]. Even tighter targets for those with comorbidities, such as diabetes or stroke disease, mean that even lower blood pressure targets are identified for older people in whom such co-morbidities are frequently present. However the average trial patient bears little resemblance to many very old people, in whom the likelihood of treatment withdrawal and hence harm, may be higher. The HYVET study, for instance, involved a physiologically robust group, with a low rate of orthostatic hypotension and no cognitive impairment [2].

People with cognitive impairment are more likely to be physically frail [9], are more likely to suffer orthostatic hypotension [10] and with age-related and arteriosclerotic changes to cerebral blood flow auto-regulation are more vulnerable to resulting cerebral hypoperfusion [11]. In addition they experience adverse events associated with polypharmacy at a lower drug count [12]. It may be, therefore, that the risk-benefit ratio of antihypertensive treatment is different in this cohort. Differing thresholds might be adopted for commencement or cessation of treatment. Many doctors believe this intuitively to be the case. Morley recently questioned whether the findings of key trials in very old people are truly generalizable, concluding that they do not apply to frail very old people and singled out blood pressure as an area where treatment targets should be attenuated [13].

Against this background we set out to describe how hypertension is currently treated in patients with dementia, whether doctors follow generic guidelines or whether they adapt their practice in light of the above considerations.

## Methods

### Search strategy and selection criteria

A pre-specified protocol was used to search for and identify suitable articles.

### Eligibility

Study characteristics: Observational studies of a population with dementia describing the prevalence of hypertension and treatments used.

Report characteristics: Non-English Language articles and studies carried out prior to 1990 were excluded.

### Information sources

A systematic search of the literature was conducted by searching electronic databases, and scanning reference lists of articles. The following databases were used: PubMed (1946 – present), Cochrane, Embase (1974 – present) and PsychINFO (1806 – present). The last full search was run on the 14th of November 2012, with updates to this until April 2013.

### Search

The following groupings of search terms were used and were adapted, as appropriate, for each database: dementia; demented; dementing; hypertension; blood pressure; antihypertensive; hypertensive; treatment; management.

An example search strategy is provided in Additional file 1.

The search was then limited to English language articles, to studies involving humans and to studies involving adults.

### Study selection

The title and abstract of the retrieved records were then assessed against the eligibility criteria in a standardised manner. Where there was uncertainty about eligibility the full article was reviewed. The bibliographies of eligible articles were searched for further relevant articles, which were again appraised against eligibility criteria.

### Data collection and items

Relevant data were extracted from the articles and entered onto a structured Microsoft® Excel database. The information sought included: (i) Characteristics of the study patients (ii) Type of study and country (iii) Prevalence of hypertension (iv) Anti-hypertensive agents used (v) Achievement of target blood pressure.

### Assessment of risk of bias

The risk of bias was assessed using the tool developed by Agency for Healthcare Research and Quality (AHRQ) [14] (Please see Additional file 2). This allowed systematic review of different potential sources of bias for each study type.

### Risk of bias

The risk of bias for each study is summarised in Table 1.

**Table 1:** Risk of bias

**Source**	**Selection bias**				**Performance bias**	**Attrition bias**	**Detection bias**			**Publication bias**	**Included in synthesis?**
	**Inclusion/exclusion criteria applied uniformly?**	**Confounding accounted for?**	**Concurrent intervention accounted for?**	**Missing data handling?**	**Outcome assessors blinded?**	**Diagnosis defined with valid and reliable measures?**	**Outcomes defined with valid and reliable measures?**	**Confounding variables assessed?**	**Outcomes pre-specified?**	**Suspicion of publication bias?**	
Amoo et al. [25]	Yes	Yes	N/A	N/A	N/A	Yes	N/A	Yes	Yes	No	Yes
Andersen et al. [26]	Yes	Yes	N/A	N/A	No	Yes	Yes	Yes	Yes	No	Yes
Davies et al. [17]	Yes	Yes	N/A	Yes	No	Yes	Yes	Yes	Yes	No	Yes
Duron et al. [20]	Yes	Yes	N/A	N/A	No	Yes	Yes	Yes	Yes	No	Yes
Hanon et al. [19]	Yes	Yes	N/A	N/A	No	Yes	Yes	Yes	Yes	No	Yes
Imfeld et al. [18]	Yes	Yes	N/A	Yes	No	Yes	Yes	Yes	Yes	No	Yes
Löppönen et al. [23]	Yes	Yes	N/A	No	No	Yes	Yes	Yes	Yes	No	Yes
Műther et al. [24]	Yes	Yes	N/A	Yes – Missing data for PWD	No	Yes	Yes	Yes	Yes	No	Yes
Poon et al. [15]	Yes	Yes	N/A	Yes – excluded	No	Yes	Yes	Yes	Yes	No	Yes
Richards et al. [16]	Yes	Yes	N/A	N/A	No	Yes	Yes	Yes	Yes	No	Yes
Rockwood et al. [22]	Yes	Yes	N/A	Yes*	No	Yes	Yes	Yes	Yes	No	Yes
Vale et al. [21]	Yes	Yes	N/A	Yes 58.8% enrolled	No	Yes	Yes	Yes	Yes	No	Yes
Zhu et al. [27]	Yes	Yes	N/A	N/A	No	Yes	Yes	Yes	Yes	No	Yes

*11.4% not contactable, 27.9% refused (community) 3.2% not contactable, 18.3% refused (institutions).

### Method of synthesis

Having extracted the data from the selected articles, the combined data was analysed to test whether there has been any change in treatment patterns over time using regression analysis. Where needed data from the articles were transformed to facilitate comparison of data.

## Results

**Figure 1:**
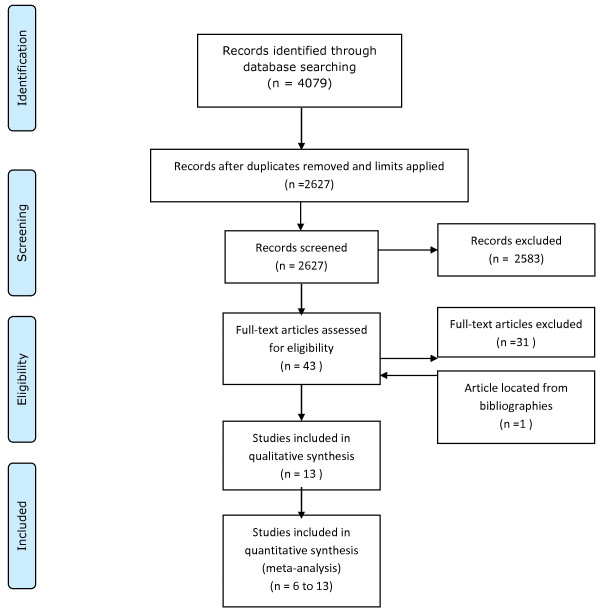
Preferred Reporting Items for Systematic Reviews and Meta-Analyses (PRISMA) flow diagram.

4079 citations were identified initially and, after applying limits and removing duplicates, this was reduced to 2627 citations. Of these 2583 articles were rejected after review of the abstract demonstrated that they did not meet the eligibility criteria. The full text of the remaining 43 articles was then reviewed in detail. 31 of these articles were then discarded after this review revealed that they were ineligible. One additional article was identified by review of the included articles’ bibliographies which met the eligibility criteria. In total, therefore, 13 articles were included in the review (Figure 1).

### Characteristics of studies

The characteristics of individual studies are summarised in Table 2. Of the 13 articles three reported studies which were conducted in the USA [15,16], two each in the UK [17,18] and France [19,20] and one each in Brazil [21], Canada [22], Finland [23], Germany [24], Nigeria [25] and Norway [26].

**Table 2:** Summary of the studies’ characteristics

**Source (year published)**	**Type of study**	**Number of people with dementia and subtypes (%)**		**Number with HTN (%)**	**Mean age (SD/range)**	**Location**	**Country**	**Identification of hypertensive pts**	**BP measured**	**Selection method**
Amoo et al. [25]	Cross-sectional	108		39 (36.1)	70	Neuro-psychiatric Hospital	Nigeria	BP > 140/90	No	All attendees with a diagnosis of dementia over a 10 year period
	Retrospective review of hospital records	AD	57							
		VaD	17							
		Mixed	4							
		Unsp.	22							
Andersen et al. [26]	Cross-sectional	187		102 (54.5)	80.9 (7)	76% community dwelling, 24% in long-term care	Norway	Self-reported medical history	No	Recent diagnosis of dementia and/or population screening. Randomly selected control group
	Case controlled	AD	100							
		VaD	0							
		Mixed 0								
		Unsp. 0								
Davies et al. [17]	Cross-sectional	20,021		9197 (46)	82.2 (7)	UK General Practice Research Database (GPRD)	UK	Having ever taken an antihypertensive for 6 months	No	Database. (Read codes for probable AD, possible AD, VaD and unspecified / other dementia searched)
	Case-controlled	AD	63							
		VaD	24							
	Retrospective	Mixed	0							
		Unsp.	13							
Duron et al. [20]	Cohort	321		221 (68.8)	78.1 (6)	Memory Clinic	France	BP > 140/90	Yes	All patients diagnosed with Alzheimer’s disease and on anti-cholinesterase treatment
		AD	100							
		VaD	0							
		Mixed	0							
		Unsp.	0							
Hanon et al. [19]	Cross-sectional	609		609 (100)	80.1 (70–86)	Community dwellers attending a memory clinic	France	BP > 140/90 or taking an antihypertensive	Yes	Consecutive attendees
		AD	86							
		VaD	14							
		Mixed	0							
		Unsp.	0							
Imfeld et al. [18]	Cross-sectional	11,524		4926 (42.7)	-	UK General Practice Research Database (GPRD)	UK	Recorded diagnosis	No	Database. (Read codes for AD, VaD and unspecified dementia + selection algorithm)
	Case controlled	AD	61							
		VaD	39							
	Retrospective	Mixed	0							
		Unsp.	0							
Löppönen et al. [23]	Cross-sectional	94		48 (51.1)	84.4 (5.7)	Population based	Finland	Recorded diagnosis or BP >160/100	Yes	All residents >65 years of age, of Lieto, were invited to take part (82% took part)
	Population based	AD	43							
		VaD	37							
		Mixed	0							
		Unsp.	20							
Műther et al. [24]	Cross-sectional	216		181 (83.8)	82.7 (6.2)	GP database	Germany	Recorded diagnosis	No	16 of 25 invited teaching GP practises. Patients with a recorded diagnosis of dementia and one of HTN, DM, hyperlipidaemia
	Case controlled	AD	0							
		VaD	0							
	Retrospective	Mixed	0							
		Unsp.	100							
Poon et al. [15]	Cross-sectional	304		304 (100)	78.1	Outpatient attendees VA medical centre clinics.	USA	Recorded diagnosis	No	Recorded diagnoses of both HTN and dementia.
	Retrospective	AD	60							
		VaD	35							
		Mixed	4							
		Unsp.	2							
Richards et al. [16]	Cross-sectional	65		37 (56.9)	81.4 (6.4)	Urban dwellers	USA	Recorded diagnosis	No	Random sample of a population derived from 29 contiguous census tracts.
		AD	75							
		VaD	0							
		Mixed	0							
		Unsp.	25							
Rockwood et al. [22]	Cross sectional	792		281 (35.5)	-	Survey of institutionalised and community dwelling older people	Canada	Recorded diagnosis	Yes	Stratified comprehensive sample across the country
		AD	57							
		VaD	26							
		Mixed	17							
		Unsp.	0							
Vale et al. [21]	Cross-sectional	186		86 (46.2)	67.4 (13.21)	Behavioural Neurology Outpatients Clinic. Tertiary referral centre	Brazil	Recorded diagnosis	No	All first attendees between 1997 and 1999 were invited to take part
		AD	31							
		VaD	19							
		Mixed	9							
		Unsp.	41							
Zhu et al. [27]	Cohort	201		71 (35.5)	76 (8.1)	84% community dwelling, 16% in long-term care.	USA	Recorded diagnosis	No	Consecutive outpatient attendees; Referrals; some long-term care residents MMSE >16, advocate available.
		AD	100							
		VaD	0							
		Mixed	0							
		Unsp.	0							

### Methodology

All 13 studies were observational studies. 11 were cross-sectional and four of these were case-controlled [17,18,24,26]. The remaining two were cohort studies [20,27]. Of the 11 cross-sectional studies, six gathered data prospectively and five did so retrospectively [15,17,18,24,25]. Of the five retrospective studies the two UK studies and the German study used databases built using data held by primary care doctors [17,18,24], and the remaining two retrospectively analysed digital and hard copy hospital data [15,25].

All of the studies described their sampling method. Six studies invited routine attendees to their clinic or hospital to take part in their study [15,19-21,25,27], three studies used information from primary care databases to identify participants [17,18,24], and four conducted population surveys [16,22,23,26].

### Participants

15,921 people with hypertension out of a total population of 23,804 were studied.

### Objectives

The objectives of the studies varied. Three set out to describe the clinical profile, including information on demographics, comorbidities and medications, of patients with dementia [21,25,27]. Four studies aimed to compare comorbidities and medication use between those with and without dementia [18,23,24,26], while one aimed to look specifically at treatment in those with vascular cognitive impairment [22]. Two studies aimed to look at the association between antihypertensives and cognitive impairment [16,17]. Two set out to evaluate the effect of antihypertensive therapy on cognitive function [19,20] and one study aimed to compare blood pressure control and medication between different ethnic groups [15].

### Individual study findings

The findings of each individual study are summarised in Table 3.

**Table 3:** Summary of the studies’ findings

**Source (year published)**	**Prevalence of HTN (%)**	**Sex (%)**	**Comorbidities (%)**		**Antihypertensive types (%)**		**Treated (%)**	**Effectiveness (meets target <140/90)**
Amoo et al. [25]	36	47 M	Heart failure	-	ACEi/ARB	-	108 (some on antihypertensives for other diagnoses)	-
		53 F	IHD	-	Diuretic	-		
			DM	6	C C Blockers	-		
			CVD	12	β-Blockers	-		
					Other	-		
Andersen et al. [26]	55	40 M	Heart failure	-	ACEi/ARB	-	125 (some on antihypertensives for other diagnoses)	-
		60 F	IHD	40	Diuretic	-		
			DM	11	C C Blockers	-		
			CVD	18	β-Blockers	-		
					Other	-		
Davies et al. [17]	46	33 M	Heart failure	-	ACEi/ARB	40	100 (population selected to be on an antihypertensive)	-
		67 F	IHD	34	Diuretic	50		
			DM	15	C C Blockers	42		
			CVD	26	β-Blockers	41		
					Other	10		
Duron et al. [20]	69	32 M	Heart failure	-	ACEi/ARB	37	78	-
		68 F	IHD	26	Diuretic	30		
			DM	9	C C Blockers	29		
			CVD	-	β-Blockers	39		
					Other	6		
Hanon et al. [19]	100	28 M	Heart failure	-	ACEi/ARB	-	55	-
		72 F	IHD	-	Diuretic	-		
			DM	-	C C Blockers	-		
			CVD	-	β-Blockers	-		
					Other	-		
Imfeld et al. [18]	43	35 M	Heart failure	9	ACEi/ARB	45		-
		65 F	IHD	22	Diuretic	90		
			DM	11	C C Blockers	45		
			CVD	-	β-Blockers	45		
					Other	-		
Löppönen et al. [23]	51	31 M	Heart failure	23	ACEi/ARB	12	85	-
		69 F	IHD	57	Diuretic	46		
			DM	18	C C Blockers	27		
			CVD	37	β-Blockers	15		
					Other	-		
Műther et al. [24]	84	23 M	Heart failure	-	ACEi/ARB	-	85	-
		77 F	IHD	-	Diuretic	-		
			DM	-	C C Blockers	-		
			CVD	-	β-Blockers	-		
					Other	-		
Poon et al. [15]	100	98 M	Heart failure	11	ACEi/ARB	59	100 (2.95) (population selected to be on an antihypertensive)	55
		2 F	IHD	31	Diuretic	57		
			DM	32	C C Blockers	44		
			CVD	19	β-Blockers	40		
					Other	20		
Richards et al. [16]	57	35 M	Heart failure	-	ACEi/ARB	25	65	-
		65 F	IHD	-	Diuretic	83		
			DM	18	C C Blockers	42		
			CVD	-	β-Blockers	8		
					Other	13		
Rockwood et al. [22]	35	29 M	Heart failure	-	ACEi/ARB	-	53	-
		71 F	IHD	-	Diuretic	-		
			DM	-	C C Blockers	-		
			CVD	-	β-Blockers	-		
					Other	-		
Vale et al. [21]	46	59 M	Heart failure	-	ACEi/ARB	-	88	-
		41 F	IHD	-	Diuretic	-		
			DM	-	C C Blockers	-		
			CVD	-	β-Blockers	-		
					Other	-		
Zhu et al. [27]	35	Not available	Heart failure	-	ACEi/ARB	-	48	-
			IHD	6	Diuretic	-		
			DM	11	C C Blockers	-		
			CVD	-	β-Blockers	-		
					Other	-		

[- = data not available].

### Synthesis of results

Data were combined from each study where available.

### Characteristics of study participants

The average age of the patients across the studies was 82, with the majority (65%) being female. Alzheimer’s disease was the most common dementia subtype (63%), followed by vascular dementia (30%), unspecified dementia (7%) and mixed dementia (0.7%). The population had a high burden of co-morbidity with 27% having ischaemic heart disease, 26% cerebrovascular disease, 12.7% diabetes mellitus and 9.3% heart failure.

### Prevalence

The prevalence of hypertension in people with dementia as reported by these studies varied between a minimum of 35% [27] and a maximum of 84% [24]. The mean prevalence of hypertension across the studies was 45% (SD 11%). There was no change in the prevalence of hypertension over time when earlier and more recent studies were compared.

### Prescribing patterns

Of the eight studies [16,19-24,27] which reported details of treatment between 48% and 85% of their participants were on at least one antihypertensive agent. Across all studies a mean of 73% were on at least one antihypertensive agent.

Diuretics (64%, range 30%-90%) were most commonly used, while calcium channel blockers (43%, range 27%-45%), ACEi/ARBs (42%, range 12%-59%) and β-blockers (42%, range 8%-45%) were less common.

A higher proportion of the hypertensive care home population took ACEi / ARBs (correlation coefficient: 0.85, R^2^ = 0.73, p = 0.031), and calcium channel blockers (correlation coefficient: 0.82, R^2^ = 0.58, p = 0.048) in later studies than in earlier studies, while the use of β -blockers and diuretics remained unchanged between later and earlier studies.

### Number of antihypertensive agents and target blood pressure

Two studies reported details of the number of antihypertensives used [15,20]. The mean number of antihypertensives was 2.4. Only one study reported on the achievement of target blood pressure [15], with 55% achieving this. This study involved 304 people, almost all male, in a Veteran Affairs hospital.

## Discussion

This review has demonstrated that hypertension is common in people with dementia and is treated in the majority. The prevalence of hypertension in people with dementia remained unchanged between older and more recent studies. Although diuretics were the most frequently prescribed antihypertensive drug, ACE inhibitors, ARBs and calcium channel blockers were prescribed more frequently in more recent studies. There was no change in the prescription of diuretics or β-blockers over this 13 year period. Only one study reported on achievement of target blood pressure, with just over half of individuals achieving this.

The studies reviewed included several large database studies, and so are likely to be representative of ordinary practice. However, they were carried out almost entirely within North America and Europe, and so the findings may not apply to countries with other health systems and prescribing habits such as in Asia or non-English speaking countries. Two of the studies reported whether participants had ever been on an antihypertensive drug rather than their current treatment. The inclusion of these data in the synthesis will have had the effect of increasing the apparent proportion on each antihypertensive class.

We are unaware of any previous systematic review looking at the treatment of hypertension in people with dementia. Similarly we are unaware of any specific guidance for the treatment of hypertension in people with dementia with which to compare these findings. Over the study period the JNC, along with others, issued a number of reports (V-VII) with changes in the generic guidance for the treatment of hypertension in older people. The rise over time in the use of ACE inhibitors / ARBs and calcium channel blockers is likely to reflect these changes in guidance. The fact that prevalence of hypertension did not change over time, despite lower blood pressure thresholds for diagnosis over time, raises the possibility that the true prevalence would have fallen over time had current, stricter, criteria for diagnosis being used throughout. It is not possible to comment from our findings whether this was the case, since several studies reported hypertension dichotomously as either present or absent using diagnostic criteria of the time, rather than presenting raw blood pressure data that we could re-analyse.

This review found no evidence that hypertension in people with dementia was not being treated. Whereas 49%-63% of people in the general US population with hypertension were on treatment [28,29], this review found that 73% of hypertensive people with dementia were on treatment. Side-effects are recognised to be a potent contributor to non-compliance in antihypertensive therapy [30,31] and the higher rates of treatment raise the possibility that side effects of antihypertensive therapy in those with dementia may be either unrecognised or unreported, in which case the favourable risk to benefit ratio observed in trials of the non-frail may not apply. In addition with theoretical concerns that, with impaired cerebral auto-regulation, this population are at increased risk of cerebral hypoperfusion [11] high rates of anti-hypertensive use, with the potential to exacerbate this, may not be ideal. Blood pressure falls as part of the natural history of dementia, starting prior to clinically apparent dementia [32] and it is intriguing that while most studies show that only 22-27% of the general hypertensive population achieve target blood pressures [28,29], 55% reached target blood pressures in the one study which reported on this in people with dementia. A recent study looking at the incidence of frailty in older patients subject to polypharmacy suggested that the use of diuretics was associated with emergent frailty, whilst ACE-inhibitors were not [33]. Thus the gradual shift, over time, to greater prescription of ACE-inhibitors may be desirable. The persistent use of diuretics may not.

## Conclusion

This review suggests that people with dementia are not managed differently from patients without dementia – despite their increased risk of adverse events and the decreased likelihood that they will be recognised or reported. The findings presented here will support further work to establish the risk-benefit of antihypertensive treatment in patients with dementia and, if differing ratios are identified, to establish dementia-specific guidelines for management.

## Abbreviations

BP: Blood pressure.

## Competing interests

The authors declare that they have no proprietary, financial, professional, or other personal competing interests of any nature or kind.

## Authors’ contributions

The literature search was designed and carried out by TJW, and the manuscript was drafted by TJW with contributions from JRG and ALG. The manuscript was critically revised by all three authors. All authors read and approved the final manuscript.

## Pre-publication history

The pre-publication history for this paper can be accessed here:

http://www.biomedcentral.com/1471-2318/14/19/prepub

## Supplementary Material

Additional file 1 Search strategy Medline (Pubmed). Click here for file

Additional file 2 Bias assessment tool. Click here for file
