# Supplementary material for: The treatment of hypertension in people with dementia: a systematic review of observational studies
Source: BMC Geriatr. 2014 Feb 12;14:19. doi: 10.1186/1471-2318-14-19 (PMC3923425; doi:10.1186/1471-2318-14-19)
Supplement: Additional file 1 — Search strategy Medline (Pubmed). [file 1471-2318-14-19-S1.docx]

Additional file 1

Search strategy Medline (Pubmed)

1. dementia
2. demented
3. dementing
4. 1 OR 2 OR 3
5. hypertension
6. blood pressure
7. antihypertensive
8. 5 OR 6 OR 7
9. management
10. treatment
11. 9 OR 10
12. 4 AND 8 AND 11
