# Supplementary material for: The treatment of hypertension in people with dementia: a systematic review of observational studies
Source: BMC Geriatr. 2014 Feb 12;14:19. doi: 10.1186/1471-2318-14-19 (PMC3923425; doi:10.1186/1471-2318-14-19)
Supplement: Additional file 2 — Bias assessment tool. [file 1471-2318-14-19-S2.docx]

**Additional file 2**

*Bias assessment tool*

Adapted from “Assessing the Risk of Bias of Individual Studies in Systematic Reviews of Health Care Interventions” a guide published by the Agency for Healthcare Research and Quality, Department of Health and Human Services, USA [^1^](#_ENREF_1).

| **Risk Of Bias** | **Criterion** |
| --- | --- |
|  |  |
| **Selection Bias** | Did the study apply inclusion/exclusion criteria uniformly to all comparison groups? |
|  | Does the design or analysis control account for important confounding and modifying variables through matching, stratification, multivariable analysis, or other approaches? |
|  | Did researchers rule out any impact from a concurrent intervention or an unintended exposure that might bias results? |
|  | If attrition (overall or differential nonresponse, dropout, loss to follow-up, or exclusion of participants) was a concern, were missing data handled appropriately (e.g., intention-to-treat analysis and imputation)? |
| **Performance Bias** | Were the outcome assessors blinded to the intervention or exposure status of participants? |
| **Attrition Bias** | Were interventions/exposures assessed/defined using valid and reliable measures, implemented consistently across all study participants? |
| **Detection Bias** | Were outcomes assessed/defined using valid and reliable measures, implemented consistently across all study participants? |
|  | Were confounding variables assessed using valid and reliable measures, implemented consistently across all study participants? |
|  | Were the potential outcomes prespecified by the researchers? Are all prespecified outcomes reported? |
| **Publication Bias** | Suspicion of publication bias? |

**References**

**1.** Viswanathan M AM, Berkman ND, Chang S, Hartling L, McPheeters LM, Santaguida PL, Shamliyan T, Singh K, Tsertsvadze A, Treadwell JR. Assessing the Risk of Bias of Individual Studies in Systematic Reviews of Health Care Interventions. In: (USA) DoHaHS, ed: AHRQ; 2012.
